# High-Resolution Motor State Detection in Parkinson’s Disease Using Convolutional Neural Networks

**DOI:** 10.1038/s41598-020-61789-3

**Published:** 2020-04-03

**Authors:** Franz M. J. Pfister, Terry Taewoong Um, Daniel C. Pichler, Jann Goschenhofer, Kian Abedinpour, Muriel Lang, Satoshi Endo, Andres O. Ceballos-Baumann, Sandra Hirche, Bernd Bischl, Dana Kulić, Urban M. Fietzek

**Affiliations:** 1 Department of Computer Science, Ludwig Maximilians University Munich, Munich, Germany; 2 Department of Electrical and Computer Engineering, University of Waterloo, Waterloo, Canada; 3 Department of Neurology and Clinical Neurophysiology, Schön Klinik München Schwabing, Munich, Germany; 4 Department of Neurology, Technical University of Munich, Munich, Germany; 5 Chair of Information-Oriented Control, Department of Electrical and Computer Engineering, Technical University of Munich, Munich, Germany; 6 Department of Neurology, Ludwig Maximilians University Munich, Munich, Germany

**Keywords:** Biophysical models, Parkinson's disease

## Abstract

Patients with advanced Parkinson’s disease regularly experience unstable motor states. Objective and reliable monitoring of these fluctuations is an unmet need. We used deep learning to classify motion data from a single wrist-worn IMU sensor recording in unscripted environments. For validation purposes, patients were accompanied by a movement disorder expert, and their motor state was passively evaluated every minute. We acquired a dataset of 8,661 minutes of IMU data from 30 patients, with annotations about the motor state (OFF,ON, DYSKINETIC) based on MDS-UPDRS global bradykinesia item and the AIMS upper limb dyskinesia item. Using a 1-minute window size as an input for a convolutional neural network trained on data from a subset of patients, we achieved a three-class balanced accuracy of 0.654 on data from previously unseen subjects. This corresponds to detecting the OFF, ON, or DYSKINETIC motor state at a sensitivity/specificity of 0.64/0.89, 0.67/0.67 and 0.64/0.89, respectively. On average, the model outputs were highly correlated with the annotation on a per subject scale (r = 0.83/0.84; p < 0.0001), and sustained so for the highly resolved time windows of 1 minute (r = 0.64/0.70; p < 0.0001). Thus, we demonstrate the feasibility of long-term motor-state detection in a free-living setting with deep learning using motion data from a single IMU.

## Introduction

Parkinson’s disease (PD) is characterized by slowness of movement, decremented small amplitude, and loss of movement spontaneity that are dramatically relieved when dopamine is orally restituted^1^. Due to the pharmacokinetic properties of the main medication, i.e. L-DOPA, motor fluctuations may occur and complicate the symptomatic treatment^2–4^. Troughs in dopaminergic therapy are accompanied by parkinsonistic phases, so-called OFF-states, while peaks can lead to phases with excessive (hyperkinetic) spontaneous movements, the dyskinetic (DYS), or ON + motor state^5^. Ideally, patients with PD (PwP) experience neither OFF nor dyskinetic motor states but maintain a state resembling normal motor function, i.e. the ON state.

These motor fluctuations are a major limiting factor for patients’ quality of life, especially in later disease stages^6^. Consequently, therapeutic innovations have to demonstrate superiority in terms of their ability to reduce motor fluctuations in order to be licensed by health agencies e.g.^7–9^. The current standard for assessing motor fluctuations relies on patient self-reporting in the form of diaries (e.g.^10^), or expert ratings using standardized scales (e.g.^11^, see^12^ for a review). Both approaches have their merits. But they are prone to rater bias and placebo effects, and they can capture the motor state only with coarse temporal resolutions^13,14^. In the past, clinically relevant features in motion data has been extracted to quantify motor states of PwP over long periods of time in free-living setups^15–17^. Those approaches were not capable of a dynamic detection of typical motion patterns and failed, for example, when the sensor data were confounded by underlying daily activities. While they achieved good correlation to the motor state at a daily scale, they erred at finer temporal resolution^18^. However, a high temporal granularity is crucial for successful and reliable adaptation of drug therapy regimes used in clinical practice, and will ultimately be essential to steer continuous closed-loop therapies such as medication pumps or deep brain stimulation in the future^19^. Despite significant technological advances in the field, there is no commonly accepted standard for objectively monitoring the motor state of PwP^20,21^. Most research on this topic has been undertaken under laboratory conditions, and only a few researchers have addressed the measurement of motor fluctuations in a free-living setting, e.g.^16,17,22–29^. While relevant progress has been shown for sensor technologies and data analyses, the critical issue of validation has not been convincingly answered^30–32^.

Recent developments in machine learning techniques (ML) such as convolutional neural networks (CNN), a class of deep feed-forward artificial neural networks^33^ have helped to analyze large-scale datasets for supporting clinical applications, e.g.^34,35^. In particular, it has been demonstrated that deep learning approaches are capable of analyzing motion data derived from sensors worn by PwP in free-living situations^25,36^. While high computational requirements for training very complex models had been a limiting factor in the past, the accessibility of high-performance computing has increasingly enabled efficient processing and interpretation of large datasets^37–39^. As most biomedical data is highly unstructured, high-quality annotations are vital for developing machine learning models; one of the major issues impacting ML applications in the clinical domain^29,40^. With additional practical, ethical, and scientific challenges including the sparse availability of annotated motion data, the lack of adequate numbers of high-quality annotations, or the non-availability of high computational power for training, ML technique has not been fully utilized for evaluating motor fluctuations of PwP in complex, free-living settings (for reviews, see e.g.^20,21,41^). Thus, we address practical challenges in free-living monitoring of the PD motor state on a larger scale, and show how ML techniques can be adapted to assist continuous monitoring of the motor state of PwP. In the present study, we used a deep learning approach, i.e. a CNN, to classify motion data from a single wrist-worn IMU sensor in a non-test-based setting. The patients were accompanied (“shadowed”) by a movement disorder expert and their motor state was passively categorized as OFF, ON, or DYS every minute. Thereby, we acquired a dataset of 8,661 minutes of annotated, cleaned and preprocessed sensor data from 30 PwP. This dataset was then trained and tested with a leave-one-subject-out (LOSO) approach to validate the CNN model’s performance. The results were compared against other models over various naturally observed ambient activities of the participants to demonstrate the robustness of the CNN performance.

## Results

### Cohort, clinical data and sensor data

The study cohort consisted of 30 patients: 20 male, 10 female. On average, the patients were 67.1 ± 10.2 years old (mean ± SD). The mean duration since the PD diagnosis was 11.0 ± 5.1 years. The mean motor score according to part III of MDS-UPDRS was 21.6 ± 15.3 (assessed in ON). The mean AIMS (sum of items 1–7) was 2.1 ± 2.4. For cohort characteristics, see Table 1. In total, 11,567 minutes of 3D-accelerometer data were collected, and 10,977 minutes of them are annotated to the expert rater’s assessment. After the pre-processing (removing data with little to no acceleration measurement as described in the Methods section), 2,316 min of data were discarded, and 8,661 min were used for further analysis.

**Table 1:** Clinical descriptors of the full cohort and according to the Hoehn & Yahr disease stages two to four.

parameter	full cohort	HY stage 2	HY stage 3	HY stage 4
	***N or mean* ± *SD (range)***			
Gender (male/female)	20/10	9/2	9/7	2/1
Age (years)	67.1 ± 10.2 (40–83)	66.1 ± 8.1 (47–73)	69.5 ± 11.0 (40–83)	58.0 ± 9.2 (48–66)
Disease duration (years)	11.0 ± 5.1 (1–21)	9.9 ± 6.0 (1–18)	11.2 ± 4.8 (2–21)	14.3 ± 2.3 (13–17)
Levodopa equivalent dose^$^	1109 ± 785 (90–3754)	1172 ± 1113 (90–3754)	1053 ± 521 (120–2435)	1181 ± 806 (675–2110)
MDS-UPDRS III (ON)	21.6 ± 15.3 (2–57)	16.9 ± 15.8 (2–57)	25.4 ± 15.6 (5–54)	19.0 ± 7.9 (13–28)
AIMS (sum items 1–7)	2.1 ± 2.4 (0–7)	1.2 ± 1.8 (0–4.5)	2.5 ± 2.7 (0–7)	2.9 ± 2.7 (1.25–6.0)
Montreal Cognitive Assessment	25.7 ± 2.8 (18–30)	26.1 ± 1.9 (24–30)	24.9 ± 3.3 (18–29)	28.0 ± 1.7 (27–30)
Body Mass Index (kg/m^2^)	25.2 ± 4.8 (12.9–35.4)	26.9 ± 5.0 (21.6–35.4)	25.2 ± 3.9 (21.3–35.1)	19.4 ± 5.8 (12.9–23.7)
Duration of motion data recording (hours)	7.5 ± 3.9 (0.4–13.4)	6.6 ± 4.0 (0.4–12.5)	8.3 ± 3.8 (2.8–13.4)	6.5 ± 5.1 (2.6–12.3)
Additional therapy, i.e. deep brain stimulation (DBS) or continuous subcutaneous apomorphine infusion (CSAI)	DBS = 4 CSAI = 2	DBS = 1	DBS = 1 CSAI = 2	DBS = 2

Mean ± standard deviation is given with the range in brackets. ^$^Levodopa equivalent dose is calculated according to ref. ^68^. Abbreviations: AIMS, abnormal involuntary movement scale; UPDRS, unified PD rating scale; DBS, deep brain stimulation; CSAI, continuous subcutaneous apomorphine infusion.

### Reliability of the expert rater

As all clinical evaluations were performed by one expert rater, we set up a separate experiment to verify the reliability of this rater (see Assessment of rater reliability). Intraclass correlation statistics of the committee vote label and the rater revealed a high intraclass correlation of 0.94 (95% confidence 0.91–0.95; F = 30.6; p < 0.0005), indicating a high consistency of our expert.

### Results from the CNN model and comparison to other machine learning models

**Figure 1:**
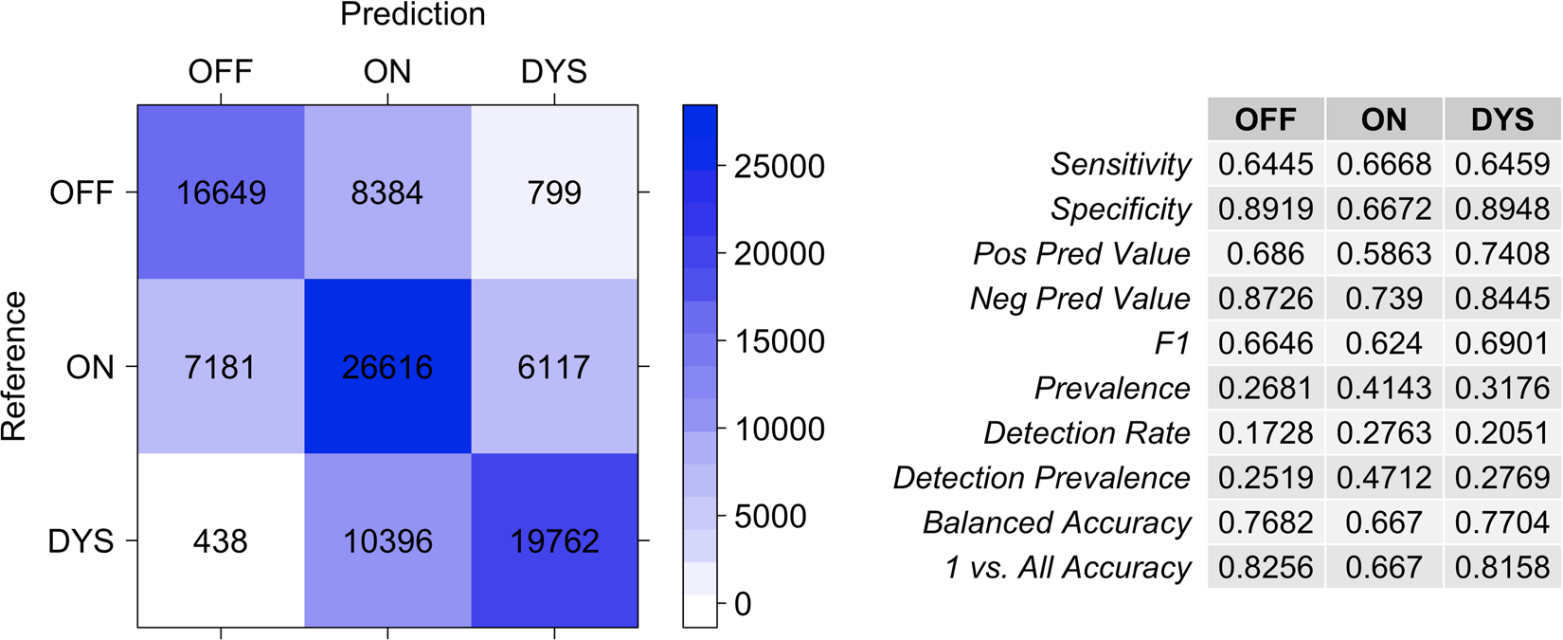
Confusion matrix of the clinical annotation (Reference) and the model prediction (Prediction), and clinimetric results of the CNN prediction for the full (augmented) dataset. The numbers in the matrix indicate corresponding observations.

The data augmentation (see Preprocessing of data)^42^ resulted in 96,342 samples of 1-minute sensor-data that had a distribution of OFF: 26.8%, ON: 41.4%, and DYS:31.8%. Our CNN model classified the three motor states of PwP at a three-class accuracy of 0.654, and a Cohen’s Kappa of 0.47, pointing to a moderate agreement^43^. Detection prevalence had a similar distribution to the true prevalence values with OFF: 25.2%, ON: 47.1%, and DYS: 27.7%, slightly overestimating the ON state. OFF and DYS were detected with better specificity compared to the ON state, with high negative predictive values (OFF: 87.2%; DYS: 84.5%). The accuracy balanced over the class distributions was highest for DYS with 0.770, compared to OFF with 0.768, and ON with 0.667. Other clinimetric results are shown in Fig. 1.

For comparisons, we implemented a SVM (support vector machine), kNN (k-nearest neighbour), Random Forest, and a MLP (multilayer perceptron) model. These models yielded consistently inferior results compared to the proposed CNN approach. These results illustrate the predictive power of the CNN-based approach in our particular dataset. See Table 2 for the detailed results.

**Table 2:** Comparison of the machine learning (ML) methods with a 4-fold cross-validation.

Methodology	SVM (linear)	kNN (n = 10)	Random Forest	MLP	CNN
Balanced Accuracy	50.28	50.41	53.73	54.02	67.39

Note that these performance measures were obtained from non-augmented data using 4-fold cross validation. Abbreviations: SVM, support vector machine; kNN, k-nearest neighbor; MLP, multi-layer perceptron; CNN, convoluted neural network.

### Temporal aggregation of probabilistic CNN output allows visualization of the motor state

**Figure 2:**
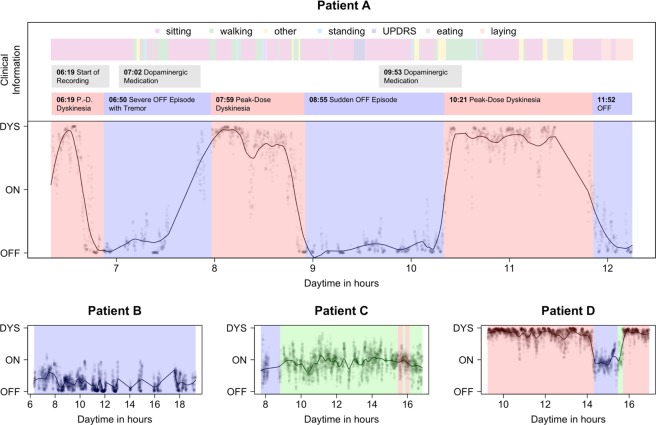
Motor state profiles of four typical patients (**A–**D). The observed motor state annotations by the expert rater is coded in three background colors along the x-axis; blue, OFF; green, ON; red, DYS). Unsmoothed expCNN point predictions (transparent circles) and LOESS smoothed predicted day curves (drawn line) predict the highly resoluted motor state. In the top section of part A, clinical information on the free-living activity is given that demonstrates the independence of the predicted motor state curve from the concurrent motor activity. Patient A is a severely fluctuating patient who spends almost no time in the ON condition, but experiences several sudden OFF or DYS phases during the recording. The expert evaluated A with an average of 1.36 ± 0.54. Mean expCNN was similar with 1.02 ± 0.81. The high SD identifies A as a severe fluctuator. Patient B is a predominantly bradykinetic patient with no ON time and no dyskinesia. B was admitted to the hospital a couple of weeks after DBS surgery and recorded before readjustment of the stimulation parameters. The expert evaluated B with an average of 0.48 ± 0.20. Mean expCNN was similar with 0.28 ± 0.22. Note that there are missing values in the predictions as the sensor signal was not recorded continuously. Patient C is a patient who is responding well to treatment. Except for the typical OFF in the morning, C spends almost all the time in the ON state. The rater evaluated C with an average of 0.99 ± 0.11. Mean expCNN was similar with 0.91 ± 0.29. The mean around 1 (=ON) and the low SD describe a patient with minor fluctuations mainly in ON state. Patient D is a dyskinetic patient with one OFF period in the afternoon. The expert evaluated him with an average of 1.47 ± 0.34. Mean expCNN was similar with 1.66 ± 0.37. The curves from all patients are available as supplementary material.

On a subject level, the aggregated clinical expert rating and the softmax scores (expCNN) were on average at a very similar level with 0.98 vs. 1.04, respectively. For easy comparison per patient, we plotted the class distribution plots of the expert ratings and the CNN predictions in an overview that can be viewed in the Supplemental Material Fig. A1. We illustrate the long-term recordings and the outcome of the algorithm for four exemplary PwP that have different clinical phenomenologies, but are equally well recognized by the algorithm (see Fig. 2 for details). We chose these specific patients to demonstrate the ability of our approach to precisely detect fast changes of the motor state.

**Figure 3:**
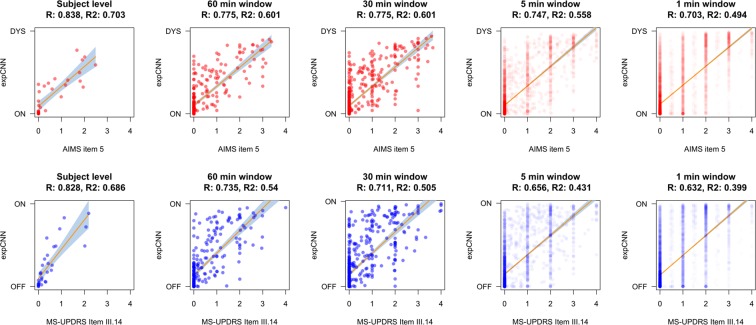
Correlations of the probabilistic output of the CNN with the bradykinesia and dyskinesia items that were labeled minutely by the expert, but modelled for various time windows. Linear correlation is depicted with 95% confidence interval (grey shading).

Figure 3 illustrates the changes in correlation between our model predictions and the clinical scores over different temporal resolutions. When the expCNN was obtained across the entire dataset per patient (i.e. a single value representing the patient’s motor state), we observe high correlations with the motor item scores of the rater, i.e. AIMS item 5 for the dyskinetic state and MDS-UPDRS item 3.14 for bradykinetic states (R_brady_ = 0.828, p < 0.001; R_dys_ = 0.838, p < 0.001). The correlation is somewhat smaller, but preserved for time windows of one hour (R_brady_ = 0.735, p < 0.001; R_dys_ = 0.775, p < 0.001), 30 minutes (R_brady_ = 0.711, p < 0.001; R_dys_ = 0.775, p < 0.001), 5 minutes (R_brady_ = 0.656, p < 0.001; R_dys_ = 0.747, p < 0.001), and also for 1-minute time windows (R_brady_ = 0.632, p < 0.001; R_dys_ = 0.703, p < 0.001).

### The model achieves similar performance for various activities

Our model is trained irrespective of background daily-life activities, and the activity annotation is not used for model training or prediction. We evaluated the performance of the CNN over the background activity classes documented by the expert rater. While being observed for their motor states, the patients spent 58% sitting, 18.5% walking, 8.4% lying, 5.3% standing, 1.2% performing motor examination tests, and 3.4% of their recording time pursuing other activities. In 5.3% of the time, the activity class was not documented. The information on patients’ activities is visualized in Supplementary Material Fig. A2. The evaluation indicated that the CNN performance was robust against the background activities of the patients as no substantial performance differences were observed between these activity classes. The CNN’s performance measures accuracy and balanced accuracy during the seven classes of activities are shown in Table 3.

**Table 3:** Prevalence and CNN performances across seven classes of background activities.

Activity (%)	Sitting	Walking	Lying	Standing	Testing	Other	Unknown
Prevalence	58.0	18.5	8.4	5.3	1.2	3.4	5.3
Balanced Accuracy	74.47	76.94	70.72	69.31	72.01	64.38	61.85

## Discussion

Our research shows that expert-level information about the motor state of PwP may be obtained from a single wrist sensor in free-living situations using deep learning autonomously at high temporal resolution. Probabilistic information and smoothing procedures demonstrate that the clinically relevant trends and the transitions from one motor state to the other are well recognized in as little as a one-minute time windows. Furthermore, we show that our method is capable of detecting the individual motor state irrespective of activities undertaken by the PwP in a free-living, non-controlled setting and generalizes to the PD population as we tested on data from previously unseen patients.

The technology empowered objectivity of this approach potentially opens new pathways for monitoring the motor state of patients with PD over the course of a day, and thus, for evaluating and developing therapies. The objective sensor-based evaluation should be more robust against measurement bias and placebo effects compared to the traditional methods such as, i.e. clinical observation by physicians or patient reported diaries. We achieved this by making use of deep learning to classify highly granular accelerometer data labeled with clinical information. The high dataset size requirements are met through a supervised data collection approach by which we were able to generate informative annotations in one-minute intervals. To our knowledge, collecting expert annotations on a one-minute basis has not been reported to date at such a large scale.

It should be kept in mind, that patients may experience their OFF states not only in terms of reduced motor function, but also in the form of pain, panic attacks, or other non-motor features^44^. We focused on the motor function, as we used a motion sensor for recordings. Other research might replicate our findings using different data types or forms of data acquisition, such as galvanic skin response or heart rate^45^. However, for standard clinical evaluation, motion analysis is currently considered the state of the art for measuring the quality of treatment^46^, and to determine the motor state.

A number of automated assessment techniques have been proposed to remotely assess motor phenomena in PwP. As recently as 2004, the detection of motor state information from body-worn sensors was considered impossible^16^. The reporting group had evaluated PwP over 1.5 hours and demonstrated limited sensitivity and specificity for tremor, bradykinesia and dyskinesia detection in comparison to self-assessment.

In this research with free-living recordings, we overcame a number of obstacles encountered by other groups. Previous work considered laboratory test setting, and evaluated patients over the course of three hours by six accelerometer sensors worn on six different body parts to distinguish ON from OFF^17^. They used statistical features of the motion data as input variables for a neural network, and reported sensitivity and specificity near 0.97. However, by essentially reducing the motor state detection to a binary classification, their results are not representative of the phenomenology of fluctuating patients. Moreover, they suggest a simple threshold to differentiate OFF from ON, that fails to be a generalizable principle. In contrast, our work shows that three motor states can be reliably differentiated.

Similarly, previous work assessed motor fluctuations from a motion sensor at the waist using an SVM for classification, and compared against 30-minute patient diaries as the ground truth^27^. Although the average specificity and sensitivity was above 0.9 for 10 min segments, these results cannot easily be transferred into clinical practice, as the algorithm did not discern ON from dyskinetic motor behavior. In addition, the use of patient reported measures for standardization is debatable, as patients are not trained to evaluate motor symptoms of PD in a systematic fashion. Moreover, there is a consensus that the motor state does not follow a 30 minute time structure, so that entries is diaries are inherently imprecise, and are a debatable source to provide valid information for the training of an algorithm. In contrast, our approach provides the means to model various time aggregations, allowing both a coarse and a granular perspective on PwP.

Another source of information regarding the motor state might be obtained from the frequency domain analysis of movement data, e.g. mean spectral power. This approach has been used to algorithmically describe bradykinesia and dyskinesia from a wrist sensor^15,47^. Although convincing group effects were demonstrated, such an approach failed to reliably provide individual predictions, and did not give information on the precise recognition of the motor state^18^. To our knowledge there is not data published using such an approach that has shown to be resistant to artificial intrusions of movement sensor recordings such as repetitive intense exercising.

Detection of motor states is highly dependent on how the task is defined. Another research group evaluated PwP over one week by patient-diary annotated 5,500 h of sensor data from bilateral wrist-worn sensors. Using a set of 91 features per minute recording, they trained a sequence of Restricted Boltzmann Machines (RBM) to classify the data in the four categories of sleep, OFF; ON; DYS^24^. They report promising specificity results for a clinical setting (0.99) and a home setting (0.93), but insufficient sensitivity below 0.6 for ON/OFF detection^25^. One might argue that framing the task of motor state detection to include sleep, that is comparatively easy to classify, leads to an overestimation of accuracy. Consequently these results are difficult to reproduce when sleep is part of the classification.

Our study was performed at one clinical center with a relatively small cohort of patients. While this research design clearly represents a limitation, it concomitantly can be interpreted as an asset.

Concerning the limitations, the small sample could be prone to investigator bias or misinterpretation, or not be representative of the large variety of the PD phenomenology or PD stages. Other sources for bias include sampling bias (e.g. rater chooses to report the motor state for a 30 minute interval from few minutes of observation, (ii) selection bias (e.g. rater describes motor state only from one motor symptom), (iii) performance bias (i.e. patient behaves better than usually) or (iv) measurement bias, i.e. pre-knowledge of outcome modifies measurement.

For these reasons, we have restricted the task to a very basic evaluation of three classes, that is expected even from the patients themselves. We evaluated the competency of our main rater by comparing his evaluation to a committee with average experience in Parkinson research of more than ten years, and found a high congruency. Future research might further improve the quality of the input to the neural networks with inclusion of aggregated voting, or using surrogate markers of bradykinesia such as the ß-band from local field recordings from the basal ganglia.

Concerning the asset, the study demonstrates the power of deep learning to recognize clinically valuable information in ubiquitously available data even in small samples^42^. Our system achieved an overall accuracy, balanced for class inhomogenities, of 65% for one-minute measurements. As all previous approaches only looked at 30 or 10 minute intervals, this accuracy stands out, and shows a promising path towards a large-scale deployment of real-life monitoring of PwP.

A major concern of motor evaluation for PwP is the choice of the patient activity during evaluation. While specific test protocols can provide for the controlled assessments of a defined function, e.g. finger taps and bradykinesia^23,48^, these results may only give a surrogate marker of the disease and may not be representative of the movement capacity of the patient^49^. On the other hand, to refrain from test setups, by relying on free-living data only, one might introduce too much noise in the dataset, thereby making any meaningful interpretation and comparison of the observations impossible.

Here, we deliberately used a free-living setting to achieve maximum ease and applicability of the approach to patients’ everyday lives. We used a commonly available smartwatch that is not licensed for a medical use and performed a limited set of data quality checks, which demonstrated reasonable data quality. Occasionally, we experienced breakdowns of the Bluetooth connection that led to recording pauses and subsequent data loss. It is conceivable that the use of more accurate and reliable devices with other sensors might further improve the outcome of our approach, but this will need confirmation in fresh datasets.

Potential sources of noise have to be discussed as well. While the sensor and rater have been evaluated for their reliability, further validation studies need to confirm our results. Also, our setup might introduce bias, as we recorded free-living data in the constrained environment of a hospital. In addition, our approach needs to be validated in a larger cohort to address the observed high inter-patient variability and potential ethnic differences between study participant groups.

Our CNN modeled the PD motor states as a 3-class categorical concept (OFF/ON/DYS) over a set of segmented time windows which are independent of each other. In order to account for the fact that patients undergo a gradual transition of classes over time, we treated the CNN prediction for each class as a confidence vector so the LOSO smoothing of aggregated softmax outputs across time could represent a continuous motor state transition. It has to be kept in mind that the confidence of the predictions is not an equivalent measure to the factual severity of the motor state but adherence to the trained model. Thus, it is adequate to interpret expCNN as a relative severity marker for the motor state rather than the absolute severity. Future research should be directed to achieve higher resolutions for severity grading, e.g. by using regression approaches. Also, taking temporal dependencies of the input into account, e.g. by adding LSTM (Long-Short-Term-Memory) layers to the deep learning models, has a potential to further improve our approach.

One important characteristic of a behavior quantification algorithm should be the capacity to describe the behavior repertoire in its totality, including behaviors not anticipated by the researchers^50^. As deep learning may be difficult to interpret, and sometimes referred to as a “black box”, the employment of visualization techniques for the convolutional filters should be investigated in the future.

We believe that the approach outlined in this paper will evolve rapidly with increasing availability of sensors and data storage capacity. For this, data security issues have to be addressed adequately. Furthermore, advances in high resolution sensor technology will demand the creation and validation of new clinical scales to more narrowly define and quantify the motor state regarding the severity and the temporal resolution.

## Methods

### Ethical vote and patient consent

This project was approved by the ethical board of the Technical University of Munich (TUM) (No. 234/16S) on June 30, 2016. All patients agreed via a written informed consent form to the protocol and to the recording and analyses of their anonymized data. The authors confirm that all experiments were performed in accordance with relevant guidelines and regulations.

### Clinical setup

Patient data was collected at the Schön Klinik München Schwabing from patients diagnosed with PD according to UK Brain Bank Criteria^51^ with an established history of motor fluctuations. The patient cohort is described by MDS-UPDRS motor score (part III)^52^, abnormal involuntary movement scale sum of items 1–7 (AIMS)^53^, Hoehn & Yahr (HY) classification^54^, Montreal Cognitive Assessment (MoCA)^55^, and body mass index during medication ON. One patient normally used a walking aid, that was not used during recording of his data.

For motor state evaluation we acquired motion data from PwP in free-living conditions, where the PwP were continuously accompanied by a movement disorder expert (D.P.) to enable large-scale annotation collection. Motion data was recorded from a sensor worn at the wrist of the more affected side. In this setting the expert provided one-minute annotations of the motor state: OFF (i.e. bradykinetic state), ON, or DYS (i.e. dyskinetic state)^56^, the severity of global bradykinesia (according MDS-UPDRS item 3.14), and of upper limb dyskinesia (according AIMS item 5). The expert only rated choreatic dyskinesias to provide annotations, as this form typically occurs during the ON motor state, while dystonic dyskinesia typically confound the OFF motor state. Therefore, if choreatic dyskinesias were observed the patient was annotated to be in a DYS motor state. For statistical comparison with the model’s predictions, the expert’s ratings for OFF, ON and DYS were considered as 0, 1 and 2, respectively. The expert also documented the current activity of the patient, i.e. sitting, lying, walking, standing, sleeping, motor testing or others. The data was collected in a continuous manner, except for short breaks due to technical reasons (such as low battery levels of the sensor) or bathroom activities of the PwP.

### Assessment of rater reliability

We acquired a second dataset consisting of 132 video segments from a different cohort of 70 PwP to allow for the evaluation of the reliability of the expert rater. Three additional movement disorder specialists and the expert rater all independently rated randomly presented short video segments according to the same classification methodology as described above. The annotations acquired by the three specialists were aggregated as committee vote labels using the mode and compared with the annotation given by the expert rater.

### Technical specifications of the sensor

The participants wore the Microsoft Band 2 (Microsoft, Redmond, WA, U.S.A.) on their wrists, as recent research identified this location best reflecting most levodopa induced variability of motor symptoms^57^. The band contains a 6-axis inertial module (LSM6D series by STMicroelectronics, Geneva, Switzerland) including a 3D-accelerometer, 3D-gyroscope, and a Bluetooth 4.0 communication module. The accelerometer records movements up to +/−8 G with a sensitivity of 0.244 mG/least significant byte and a sampling rate of approx. 62.5 Hz.

### Preprocessing of data

The 11,567 minutes of collected accelerometer data was preprocessed to classify the motor state. As the raw data includes sensor noise, we mildly filtered by applying a two-directional Butterworth filter. The lower bound of the cut-off frequency was set to 0.1 Hz to filter out sensor drift, and the upper bound was set to 20 Hz to filter out high frequency noise. The sensor data was resampled to 60 Hz to create regular intervals between samples. Accelerometer data with no clinical annotations (590 min) and ones with little to no signals (a sum of variance of x, y, z accelerations less than 0.01, 2316 min) were removed, resulting in a dataset of 8,661 minutes. Note that only the three Cartesian dimensions of acceleration are used because PD motor states are believed to be independent from the pose of the wrist. As a result, each one-minute window of data is a 3600 * 3 matrix, that will be regarded as a rectangular image input for the CNN. After resampling and cleaning of the data, the data was augmented by rotation and sliding-window-based data augmentation methods^42^. Rotational augmentation emulates different arm poses, which may be present independently of the PD motor states, by applying a random rotation to each 1-min windowed data. On the other hand, sliding-window-based augmentation extracts multiple 1-min windows from consecutive two 1-min windows by sliding the window with a stride of 5 seconds. Data augmentation increased the size of the data from 8,661 to 96,342 samples. In cases where the sliding window was located in the middle of two differently annotated 1-min windows, we used float annotation after linear interpolation.

### Comparison to other classification methods

We implemented a set of classical machine learning methods including SVM (support vector machine), kNN (k-nearest neighbor), Random Forest, and MLP (multilayer perceptron) using Scikit-learn and Keras to serve as comparative models to our approach. The performance of these models was evaluated using a 4-patient-group cross-validation procedure where the dataset was divided into four similar-sized patient groups. Note that all methods are applied to the raw data without any feature extraction steps.

### Architecture, development and tuning of the CNN

**Figure 4:**
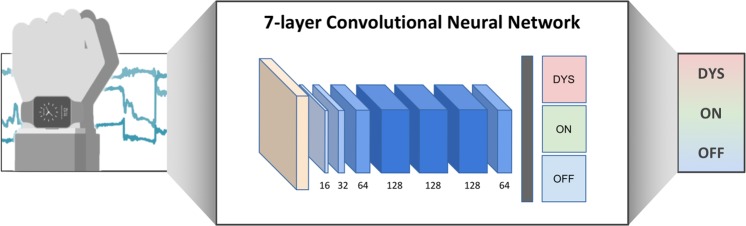
Setup of IMU sensor data acquisition and CNN architecture. The CNN consists of seven convolutional blocks followed by two fully-connected layers. Each block consists of a convolution, batch-normalization^66^ and ReLU^67^ layer. As we progress through the convolutional blocks, the size of the input vector decreases from 3600 * 3 to 13 * 1, whereas the number of channels increases from 1 to 64. Finally, the 13 * 1 * 64 feature maps are flattened to an 832 * 1 vector and classified by two fully-connected layers which have 512 and 3 nodes, respectively. (Figure drawn by Dr. Pfister).

CNNs were originally developed for processing 2D images (e.g.^33,58^). However, CNNs have also shown powerful performance in non-image data by treating them as 2D images, e.g. for Natural Language Processing (NLP)^59^, or wearable-sensor data^60^. In this research, an ensemble CNN approach was used for classifying the three motor states. Each CNN in the ensemble CNN consists of 7 convolutional layers with 64-128-256-512-1024-1024-1024 feature maps. Each layer again consists of convolution (Conv), batch-normalization (BN), and rectification (ReLU) sublayers. The architecture of the CNN is depicted in Fig. 4.

Each CNN in the ensemble was trained with a 15-randomly selected subset of patients, to handle differing motor state distributions between patients. The input window size included one-minute sequences of sensor data with a sliding window of five-second length. The sensor data from each one-minute snippet was transformed to a three-channel RGB image where each sensor corresponded to one channel. For each sensor stream, 3600 data points were depicted as the sensors operated with a frequency of 60 Hz. Those images were fed as inputs to the CNN^61^. For a more detailed description of the transformation method, see^36,62^.

### Postprocessing of CNN output

The three output nodes of the CNN normalized by the softmax function are considered as probabilities of the output belonging to each of the respective three classes OFF, ON, DYS. Thus, in order to visualize the CNN output, two mathematical operations were performed on the CNN output:

(i) Aggregation of softmax output to compute expCNN ranging from 0 (OFF) via 1 (ON) up to 2 (DYS):
  $${\rm{expCNN}}=0\ast \text{softmax}(0)+1\ast \text{softmax}(1)+2\ast \text{softmax}(2)$$
(ii) Visualization of expCNN by means of LOESS smoothing^63^.

It should be noted that the magnitude of expCNN does not directly reflect motor symptom severity, but a probability of belonging to the three classes: OFF, ON, DYS.

### Comparison of rater and CNN output

We employ a LOSO cross validation (CV) strategy which is the classical exhaustive CV procedure for small datasets^64^. This CV strategy evaluates how the trained model generalizes to data from participants not seen during training. That is, in each fold, one participant contributes all observations of the test data, while the CNN is trained with data from the remaining participants. This strategy provides a more realistic test of the system performance, and is more similar to the deployment where we would have little opportunity to create a training dataset for every new user^65^. As the task was a multi-class classification problem, a 1-vs-all strategy was used for the calculation of the clinimetric results shown in Fig. 2. For visualization of the variation in time, the expCNN output is plotted with the actual motor state derived from the expert rater’s ground truth three-class annotations (see Fig. 2 for examples).

For demonstration of criterion validity, the rater’s mean item values for bradykinesia and dyskinesia were correlated with expCNN (0 to 1 for bradykinesia, 1 to 2 for dyskinesia) as has been done in related research^15^. Also see Fig. 3. To determine the reliability of our approach across various temporal granularity, we used time windows of 1 min, 5 min, 30 min, and 1 hour – in addition, we also aggregated on the whole recording per subject (daily window).

### Patients’ view on experiment

To determine the patients’ acceptance of the experiment, a short survey with four questions was conducted after the experiment. The results are presented in the Supplementary Material Fig. A3.

### Descriptive and computational statistics

Group comparisons of clinical characteristics are performed using a one-way ANOVA. Correlations are calculated in terms of Pearson’s correlation coefficients with 95% confidence limits for mean and single predictions. Interrater reliability of the expert was ascertained with intraclass correlation statistics (ICC 2,1). Alpha was 5%. Statistical analyses and machine learning were performed with R, version 3.4.2 (R Project for Statistical Computing) and Python, version 3.5 (Python Software Foundation) employing Keras and Tensorflow for its backend. Deep learning models were trained using a GPU, GTX 1080ti (12GB) by NVIDIA, Santa Clara, USA.

## Supplementary information

Supplementary Material.

## Data Availability

The patients of this proof-of-concept study, in accordance to National and European Data protection laws, did not consent to publication of their sensor data in open repositories. The data that support the findings of this study are available from the corresponding author, U.M.F., upon reasonable request.

## Code availability

We made the code for the CNN publicly available. The repository can be found here. https://github.com/terryum/Deep_Ensemble_CNN_for_Imbalance_Labels. In order to run the code, we provide a modified dataset based on MNIST (http://yann.lecun.com/exdb/mnist/).
