# Supplementary material for: High-Resolution Motor State Detection in Parkinson’s Disease Using Convolutional Neural Networks
Source: Sci Rep. 2020 Apr 3;10:5860. doi: 10.1038/s41598-020-61789-3 (PMC7125162; doi:10.1038/s41598-020-61789-3)

Supplemental Material

High-Resolution Motor State Detection in Parkinson's Disease Using Convolutional Neural Networks

Franz MJ Pfister[1], Terry Taewoong Um[2], Daniel C. Pichler[3,4], Jann Goschenhofer[1], Kian Abedinpour[3,4], Muriel Lang[5], Satoshi Endo[5], Ruth Adam[6], Sandra Hirche[5], Andres O Ceballos-Baumann[3,4], Bernd Bischl[1], Dana Kulić[2], and Urban M Fietzek[3,6*]

[1] Computational Statistics, Department of Statistics, Ludwig Maximilians University, Munich, Germany
[2] Department of Electrical and Computer Engineering, University of Waterloo, Waterloo, Canada
[3] Department of Neurology and Clinical Neurophysiology, Schön Klinik München Schwabing, Munich, Germany
[4] Department of Neurology, Technical University of Munich, Munich, Germany
[5] Department of Electrical and Computer Engineering, Technical University of Munich, Germany
[6] Department of Neurology, Ludwig Maximilians University, Munich, Germany

* corresponding author

Supplemental Figure A1

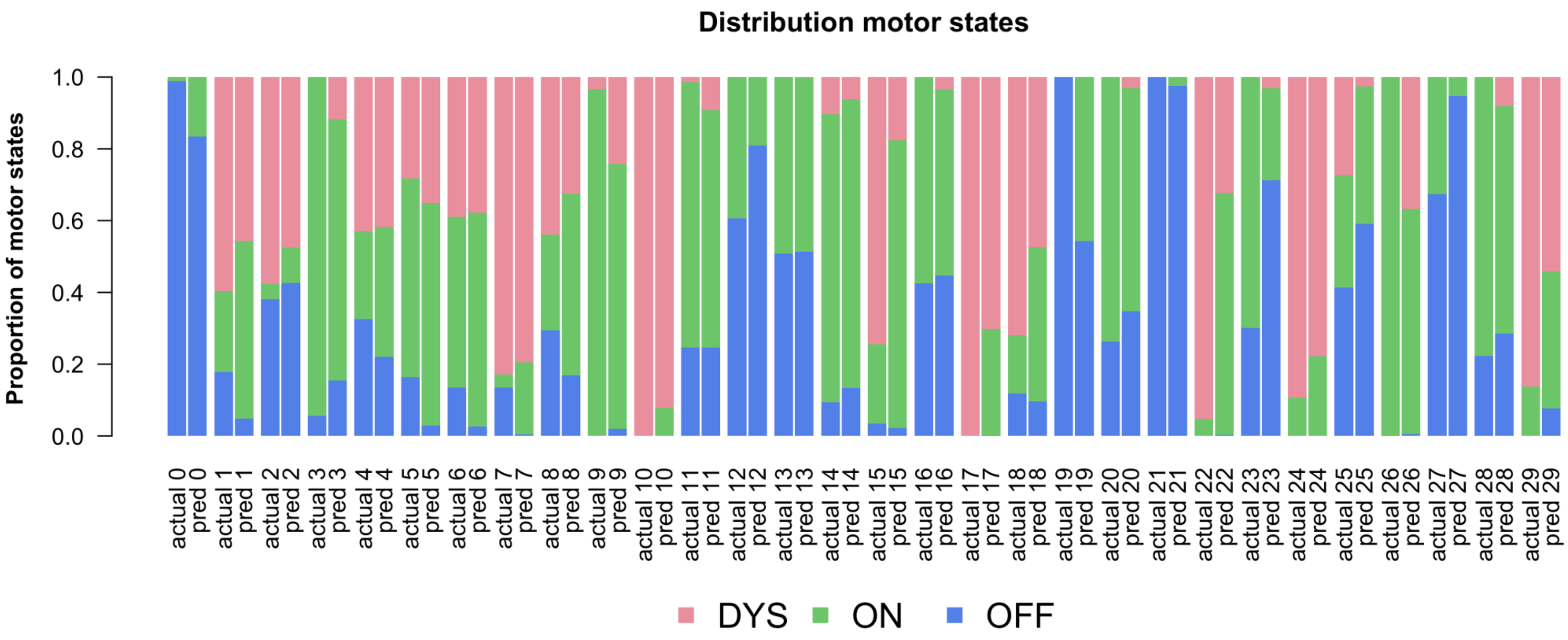

Legend

Distribution of accumulated time spent in the different motor states over all 30 patients. Depicted are the rated (actual), and the predicted motor states per patient. Note that the individual character of every patient is preserved in the algorithmic prediction.

Supplemental Figure A2

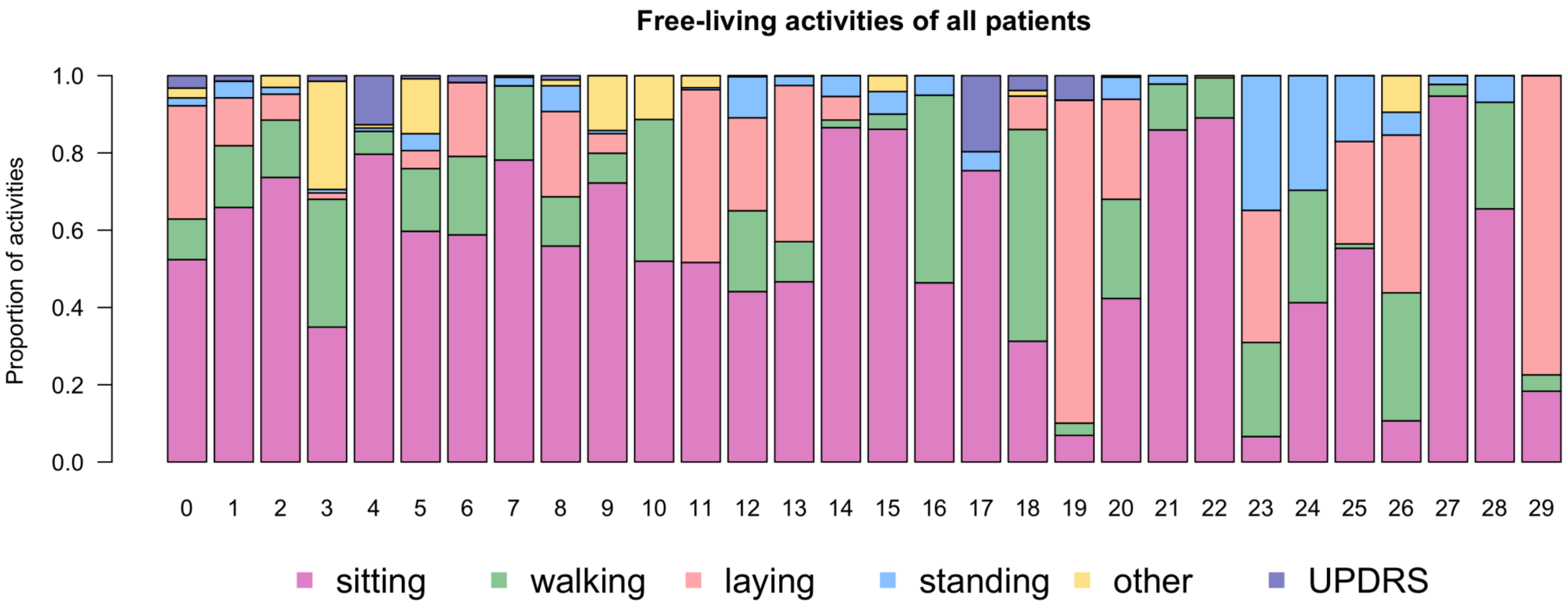

Legend
Stacked columns demonstrate the relative contribution of various activities to the free-living recordings of every single patient.

Supplemental Figure A3

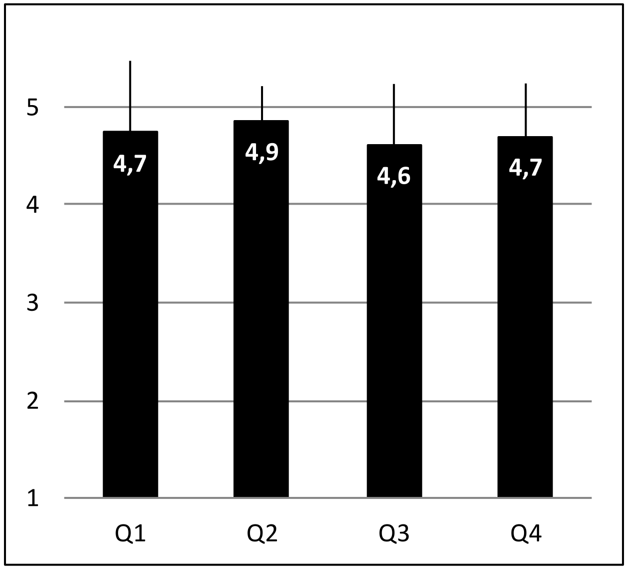

Legend
Patients were to comment after the recordings on these four statements by a Likert-type scale:
1, fully disagree; 2, partially disagree; 3, undecided; 4, partially agree; 5, fully agree.

- Q1: The sensor band did not bother my activities of daily living.
- Q2: The band was easily instrumented.
- Q3: I can imagine to wear a sensor band at home.
- Q4: I can recommend to other patients to wear a sensor band.

The columns show mean values, antennas signify standard deviation.

Supplemental Figures B1-B30

Legend

Motor state profiles of all 30 patients.

The figures are analogues to Figure 3 in the paper.

The motor state as labeled by the expert is coded in background blocks with three colors along the x-axis; OFF, blue; ON, green; DYS, red.

Unsmoothed expCNN point predictions (transparent) and smoothed motor state curves (solid line) demonstrate the predicted motor state.

Figures B1-B4

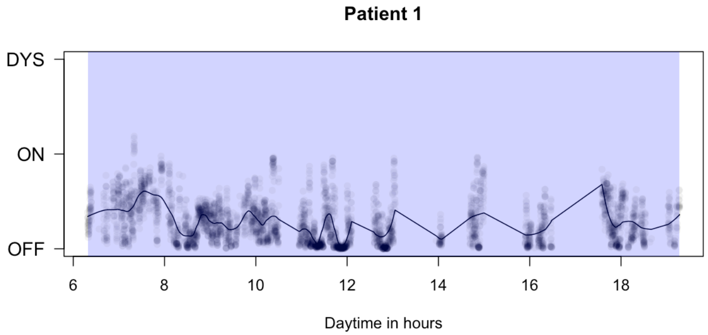

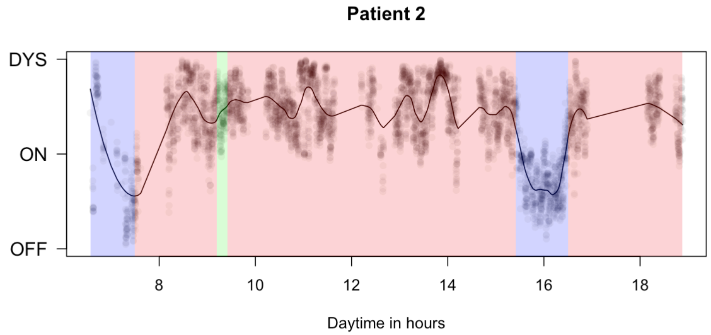

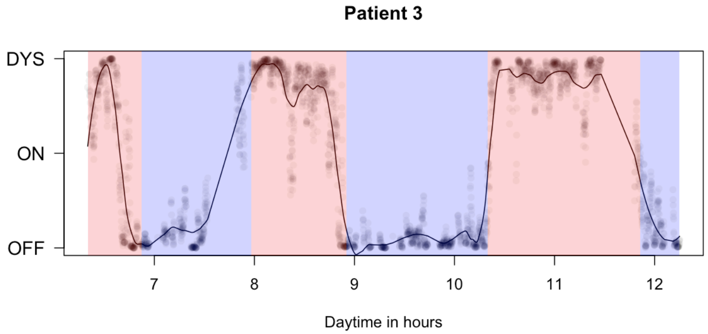

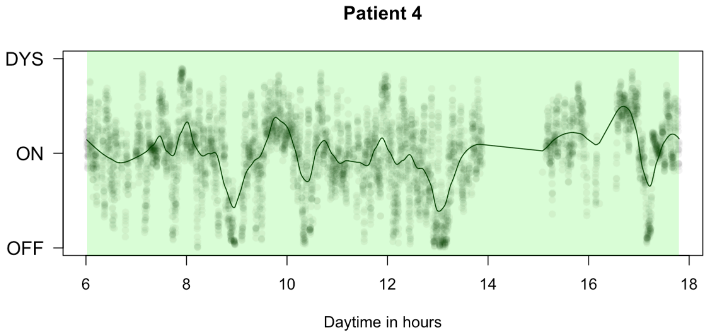

Figures B5-B8

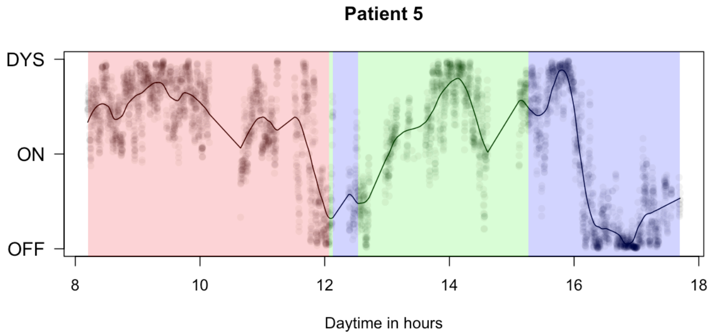

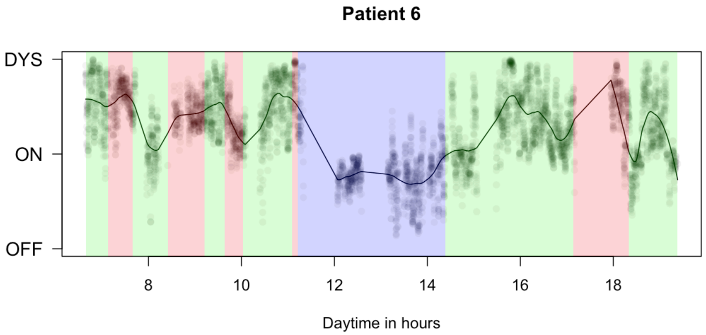

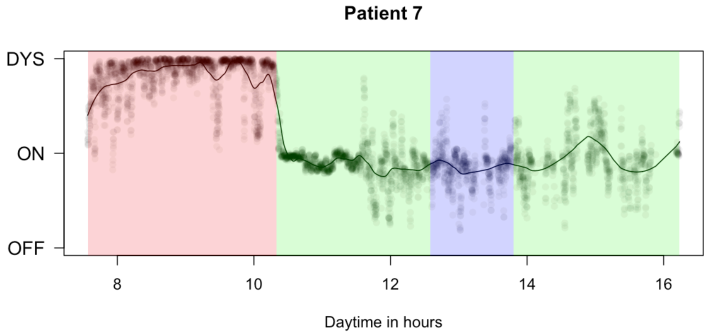

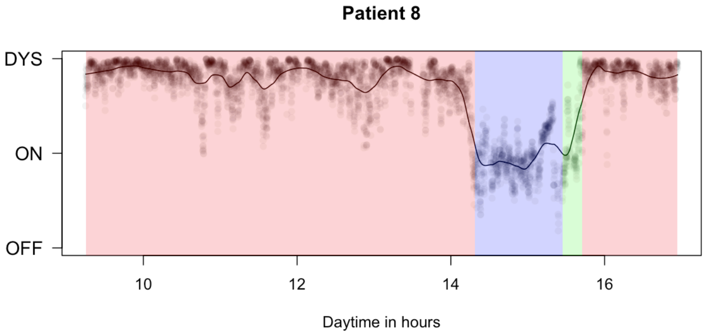

Figures B9-B10

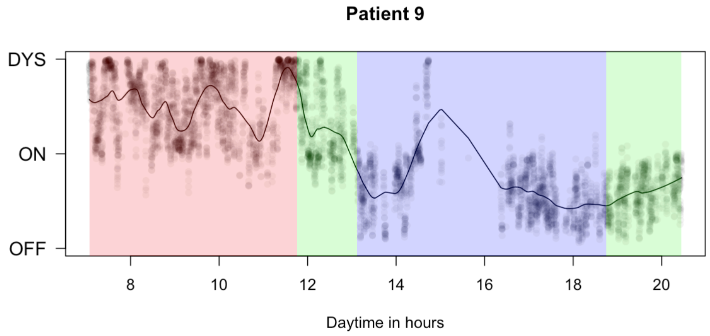

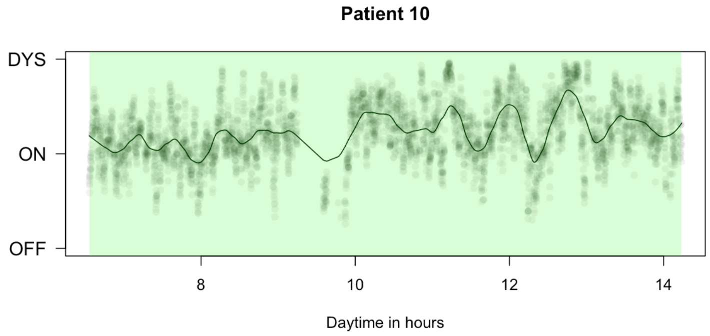

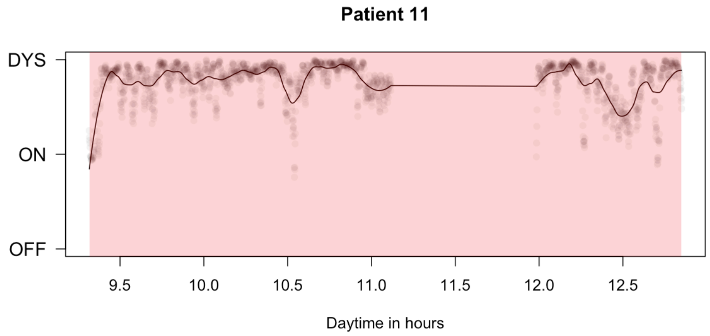

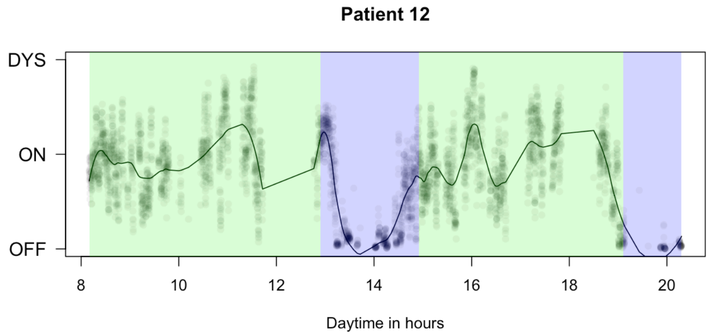

Figures B13-B16

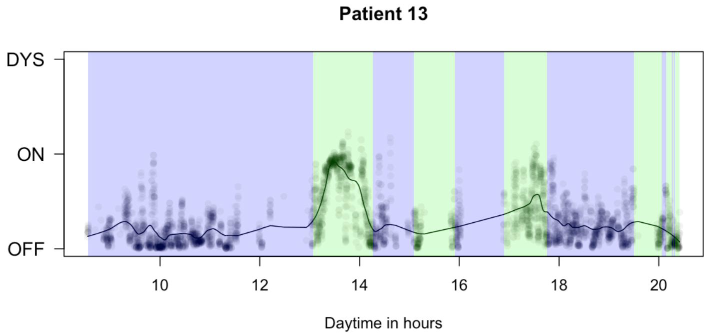

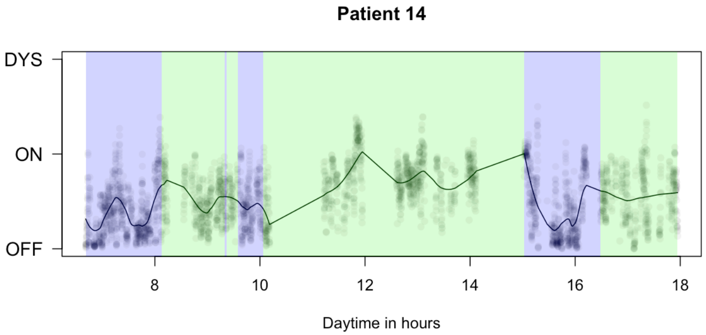

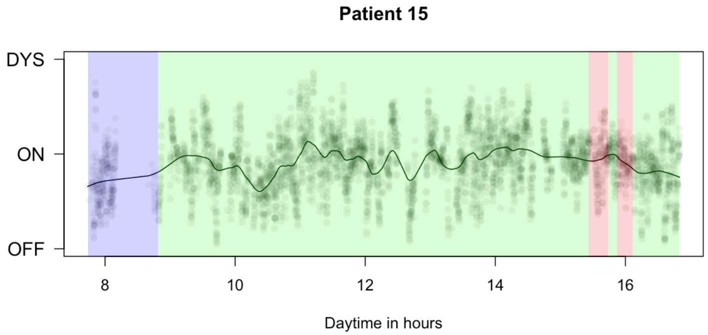

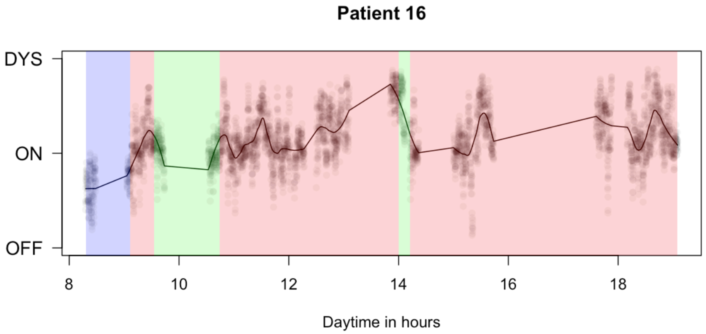

Figures B17-B20

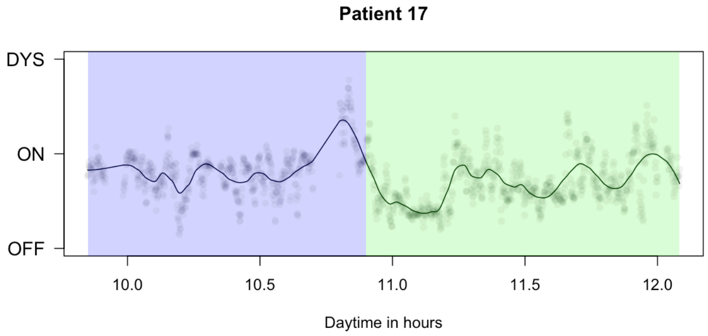

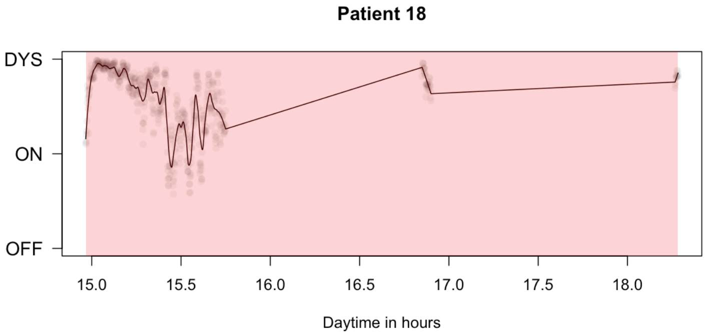

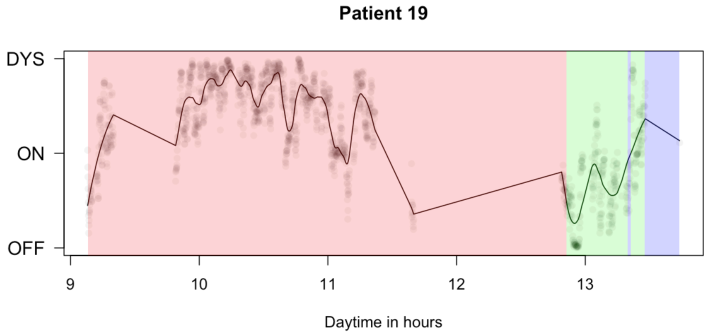

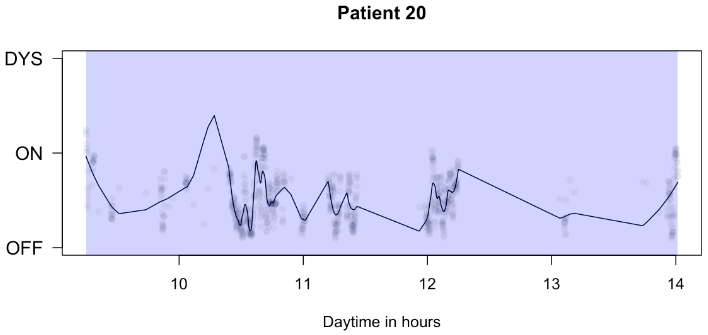

Figures B21-B24

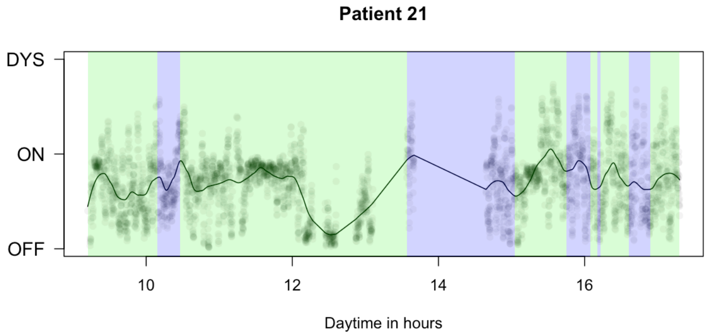

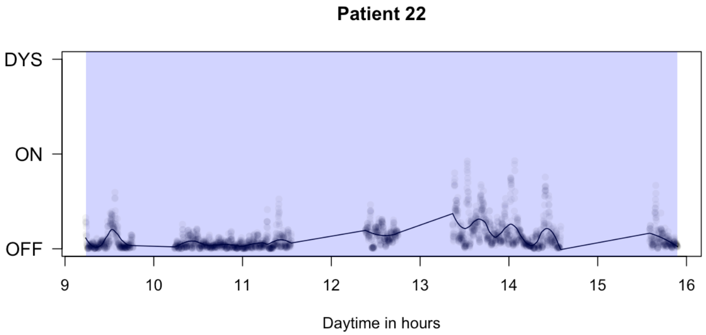

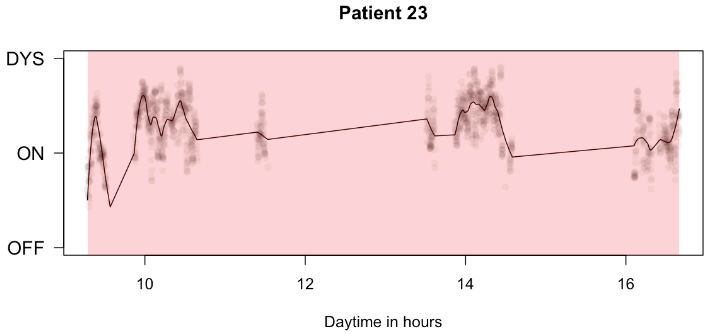

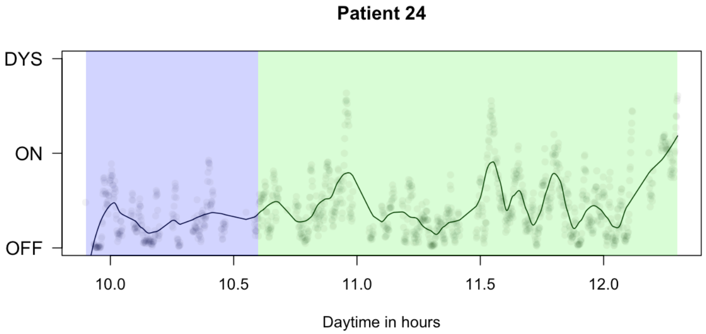

Figures B25-B28

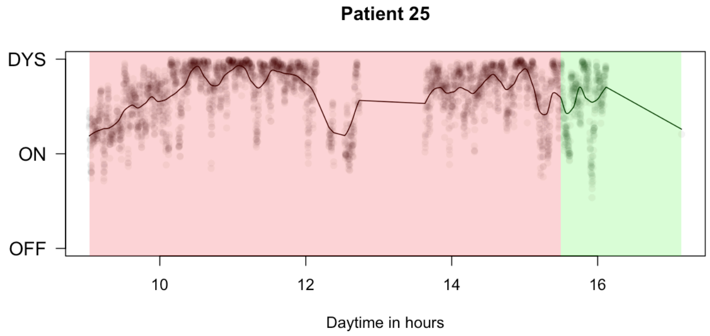

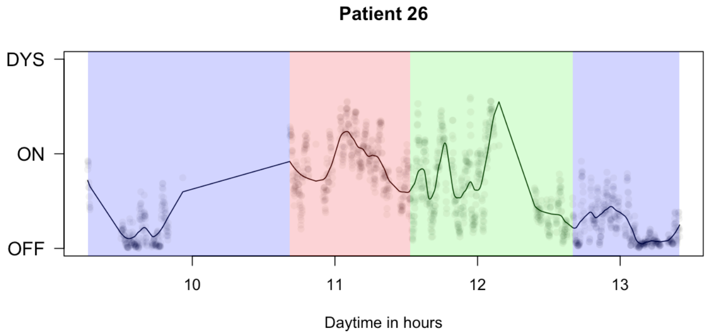

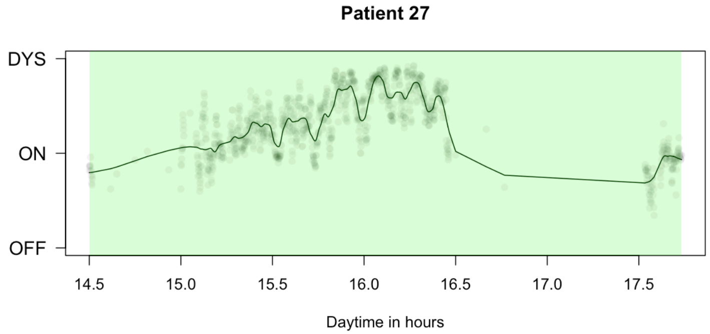

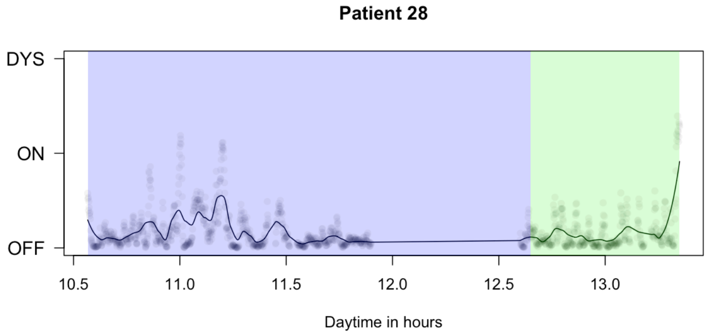

Figures B29-B30

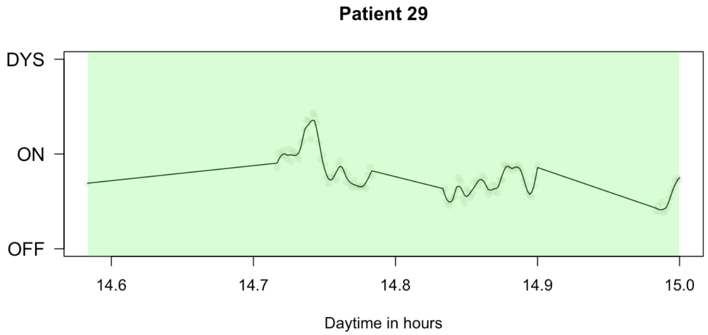

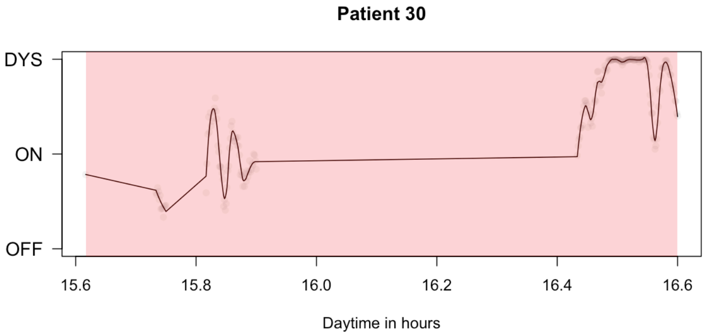

Supplement: Supplementary file 1 — Supplementary Material. [file 41598_2020_61789_MOESM1_ESM.pdf]
